# Development of a New Tool for 3D Modeling for Regenerative Medicine

**DOI:** 10.1155/2011/236854

**Published:** 2011-06-13

**Authors:** Filippo Mattoli, Roberto Tiribuzi, Francesco D'Angelo, Ilaria di Girolamo, Mattia Quattrocelli, Simona Montesano, Lucia Crispoltoni, Vasileios Oikonomou, Maria Gabriella Cusella De Angelis, Peggy Marconi, Antonio Orlacchio, Maurilio Sampaolesi, Sabata Martino, Aldo Orlacchio

**Affiliations:** ^1^Dipartimento di Medicina Sperimentale e Scienze Biochimiche, Sezione di Biochimica e Biologia Molecolare, Università degli Studi di Perugia, Via del Giochetto, 06126 Perugia, Italy; ^2^Translational Cardiomyology Laboratory, SCIL K.U. Leuven, 3000 Leuven, Belgium; ^3^Dipartimento di Medicina Sperimentale, Sezione Anatomia Umana, Università di Pavia, 27100 Pavia, Italy; ^4^Dipartimento di Medicina Sperimentale e Diagnostica, Sezione di Microbiologia, Università degli Studi di Ferrara, 44121 Ferrara, Italy; ^5^Laboratorio di Neurogenetica, CERC-IRCCS Santa Lucia, Rome, Italy; ^6^Dipartimento di Neuroscienze, Università di Roma “Tor Vergata”, Rome, Italy

## Abstract

The effectiveness of therapeutic treatment based on regenerative medicine for degenerative diseases (i.e., neurodegenerative or cardiac diseases) requires tools allowing the visualization and analysis of the three-dimensional (3D) distribution of target drugs within the tissue. Here, we present a new computational procedure able to overcome the limitations of visual analysis emerging by the examination of a molecular signal within images of serial tissue/organ sections by using the conventional techniques. Together with the 3D anatomical reconstitution of the tissue/organ, our framework allows the detection of signals of different origins (e.g., marked generic molecules, colorimetric, or fluorimetric substrates for enzymes; microRNA; recombinant protein). Remarkably, the application does not require the employment of specific tracking reagents for the imaging analysis. We report two different representative applications: the first shows the reconstruction of a 3D model of mouse brain with the analysis of the distribution of the *β*-Galactosidase, the second shows the reconstruction of a 3D mouse heart with the measurement of the cardiac volume.

## 1. Introduction

Regenerative medicine-based applications represent a promising therapeutic approach for diseases with degenerative tissues as hallmarks (i.e., Alzheimer's disease, lysosomal storage disorders, and some cardiac pathologies). To this end, innovative combined gene transfer/stem cell implantation strategies are advanced in order to reestablish the genetic defect as well as the damaged tissues/organs [1–7]. Parallel effort is also made on the development of new tools able to investigate the tissues/organs architecture after treatment [8–13]. Thus, the three-dimensional (3D) computational reconstruction of anatomical tissues/organs following surgery represents one of the necessary analytical instruments. 

The most common technique for creating such models is based on the 3D reconstruction from serial cross-section images collected through several conventional techniques (Computerized Tomography, Positron Emission Tomography, Magnetic Resonance Imaging, 3D ultrasound, and X-ray) as well as by synchrotron radiation or diffraction-enhanced imaging [12–17]. However, these techniques do not supply information on the macromolecular composition in the image. Conversely, spectroscopy-based techniques (i.e., 3D IR-imaging) present the advantage of underlying the image macromolecular composition directly [18–20], but, because of the lack of penetration of mid IR radiation into the tissue, this method precludes a real-time imaging of whole samples. Advancing in computational technology (open source library (e.g., http://www.eecs.tufts.edu/~alauri02/install.htm) [20, 21] as well as open source software (e.g., http://www.fas.org/dh/)) support the overall imaging analysis procedures and the development of new tools of imaging investigations. In this contest, progress will come from computational methods able to detect target molecules of various compositions within tissues/organs.

Addressing this issue, we present a simple and usable framework that does not require sophisticated or expensive apparatus. The procedure integrate 2D images obtained by collecting serial tissue/organ slices into a 3D computational model but is finalized to analyze the presence and distribution of target molecules as well as other tissue/organ parametric characteristics. The main advantage of our method is that it is applicable to postmortem analysis of samples processed in every experimental condition. The application resolves some limitations of the above described techniques since together with the anatomical structure information of the reconstructed tissue/organ the method allows the real-time detection of target macromolecules. 

Here, we reported two different applications of our computational procedure. The first consists in the generation of a 3D brain model using C57/BL6 mice after *in vivo* gene transfer with HSV-T0Z herpes simplex viral vector. Mainly, we present the reconstruction of the 3D distribution of the transgene within the brain. The second consists in the generation a 3D model of C57/BL6 mouse heart in order to measure the cardiac volume values. 

## 2. Materials and Methods

### 2.1. Materials

C57/BL6 mice were from Charles River, Italy. The 5-bromo-4-cloro-2-indlyl-*β*-D-galactopiranoside (X-Gal) was obtained from Sigma Chemical Co. The medium for tissue culture was from Euroclone, Celbio Lab., fetal calf serum was from Mascia Brunelli, penicillin/streptomycin was from Gibco BRL. All reagents used in this study were of analytical grade. 

### 2.2. HSV-T0Z Viral Vector Direct Injection into the Mice Brain

One group of 5-month-old animals was injected with a dose of herpes simplex viral vector encoding for the *β*-Galactosidase enzyme (5 × 10^6^ PFU, HSV-T0Z) into the internal capsule of the left brain hemisphere of the mice as previously described [22]. Mice were anesthetized with 0.02 mL/g body weight of 2,2,2-tribromoethanol and 2-methyl-2-butanol and placed on the Styrofoam platform of a stereotaxic injection apparatus (David Kopf Instruments, Tujunga, Calif, USA). The skull was exposed following a 10 mm incision in the midline. The injection coordinates for the internal capsule were −0.34 mm to bregma, 1.4 mm mediolateral, and 3.8 mm depth. These coordinates were chosen in order to minimize vector leakage into the ventricular space. Each injection was 5 *μ*L total, and the injection speed was 0.1 *μ*L/min. The injections were carried out using a needle capillary (1.2 mm × 0.6 mm) attached to a Hamilton syringe. The injections were delivered at a rate of 0.1 *μ*L/min, and the needle was slowly withdrawn after an additional 5 minutes. The scalp was closed by suture.

All procedures were performed according to protocols approved by an internal animal care and use committee and were reported to the Ministry of Health, as per Italian law.

### 2.3. Brain Serial Section Preparation

One month after injection, mice were sacrificed by cardiac perfusion. The left ventricle was cannulated, an incision was made in the right atrium, and the animals were perfused with 2% paraformaldehyde in PBS until the outflow ran clear, then the brain was included in ornithine carbamoyl transferase (O.C.T. compound, Tissue-Tek, Sakamura, The Netherlands) after exposure to 5%–30% glucose gradient and finally sectioned on a cryostat into 15-*μ*m-thick serial sections. 

We collected brain serial sections in four series of slides (A-B-C-D), so that: section 1 on slide B3 was collected immediately after section 1 on slide A3 and immediately before section 1 on slide C3. Thus, staining only 1/4 of the sections (A), we checked the beta-gal staining distribution (1 section every 60 *μ*m) along the whole brain extension.

After perfusion, whole spines were removed, and after decalcification in 3% Trifluoracetic acid (Merck), spinal cords, surrounded by vertebrae and remains of skeletal muscles, were cut into blocks containing a known number of vertebrae (four or five). Each block was sectioned on a cryostat into 10 *μ*m serial sections. 

Animal experimentation protocols were approved by the Italian ethical committee.

### 2.4. Galactosidase Analysis

*β*-Galactosidase activity was assayed through the histological substrate X-Gal as previously described [22].

### 2.5. C57/BL6 Hearts Sectioning

For histological heart analysis, C57/BL6 mice were sacrificed with an intravenous bolus injection of saturated potassium chloride, aiming at inducing cardiac arrest at systole, and the hearts were rapidly excised for fixation in buffered formaldehyde (4%), washed in PBS and quickly frozen in OCT. Serial heart sections were fixed with 4% PFA, and processed for hematoxylin-eosin staining according to standard procedures. Images were taken with a S100 TV microscope (Carl Zeiss MicroImaging Inc.) or with deconvolution microscope DeltaVision RT for the panelling image.

### 2.6. Development of Routines

The routines were developed in Visual Basic 6 and applied on images of format “.bmp” acquired by microscope. 

#### 2.6.1. To Reduce Image Soils and Glares

**Figure 1:**
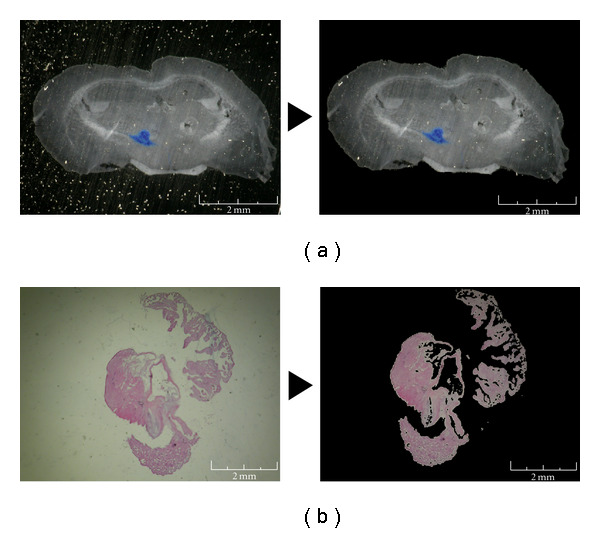
Soil and glare reduction. Soils and glares have been removed in order to have a clear vision of the final model. (a) We have on the left the original image of the section of brain tissue, and on the right the image after soil and glare reduction. (b) The same procedure has been used for heart section images.

The routine clears images, transforms to black colour (RGB 0,0,0) any pixel with intensity of red/green/blue comprised between the values chosen by customers. Customer can select values by dragging windows in tissue and background areas. The software allows separating foreground from background. In particular, salt/pepper noise may be eliminated through the selection of colour levels adequately. The output images will contain only the tissue colour range (Figure 1). We did not modify the images. We have always converted the background into black colour. This step is based on the selection of the colour tonality that is already part of the background. 

#### 2.6.2. To Centre the Image

The routine centres the section area in the image elaborating each file by moving the selected area from the total amount:

(1) $$\frac{\sum_{n}^{1}x_{n}}{n}-x_{0},\;\;\frac{\sum_{m}^{1}y_{n}}{n}-y_{0}.$$

“*n*” is the number of not black pixels (not RBG 0,0,0), “*x*_*n*_” and “*y*_*n*_” are coordinates of these pixels, “*x*_0_” and “*y*_0_” are the centre of bitmap coordinates. 

**Figure 2:**
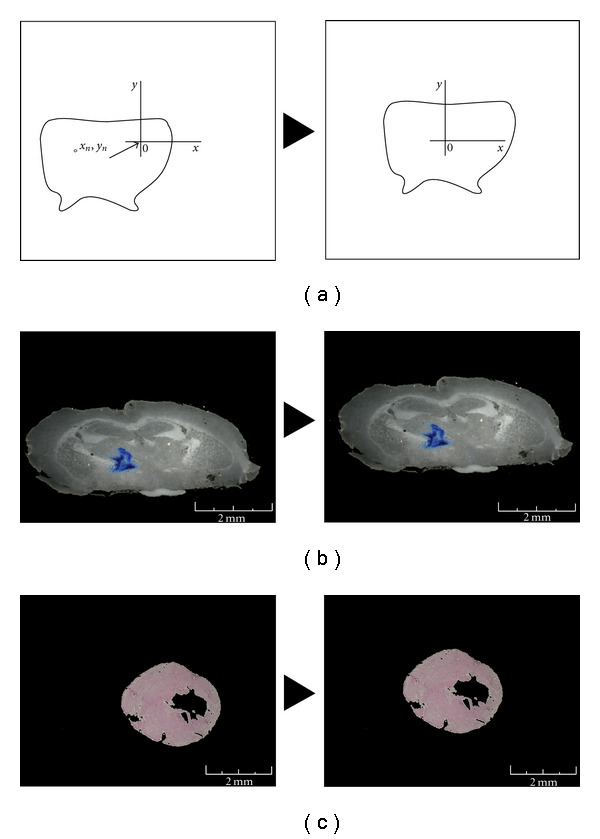
Woven image centre. (a) Outline (the reported image doesn't represent any specific tissue section) of the image centring routine (see routine 1). *x*_*n*_  and *y*_*n*_ are coordinates of not black pixels (not RBG 0,0,0), *x*_0_ and *y*_0_ are the centre of the bitmap coordinates. The program calculates a kind of barycentre of the woven area in the image and moves it to the centre of the bitmap. (b) Example of the image centring procedure: left panel is an original image of the coronal serial sections, right panel is the same image after the automatic woven centring. (c) Example of the image centring procedure for heart section images.

The centering step does not require foreground center of each slice. In fact, even in the case where the slice section is deformed, the method guarantees a correct analysis (Figure 2).

#### 2.6.3. To Orientate the Image

The routine orients the image of each section with respect to the adjacent section by rotating the tissue area of the bitmap 360°, and for every fraction of rotation calculates the difference “*d*”:

(2) $$d=\overset{1}{\underset{n}{\sum }}\left(i_{n}-k_{n}\right).$$

“*i*” is the intensity of the pixel “*n*” of the first image and “*k*” is the intensity of the pixel “*n*” of the previous image. Notably, the section orientation may be validated by comparing the anatomic tissue structure of the slice section using specialized mouse atlas as reference (e.g., http://www.bnl.gov/CTN/mouse/).

**Figure 3:**
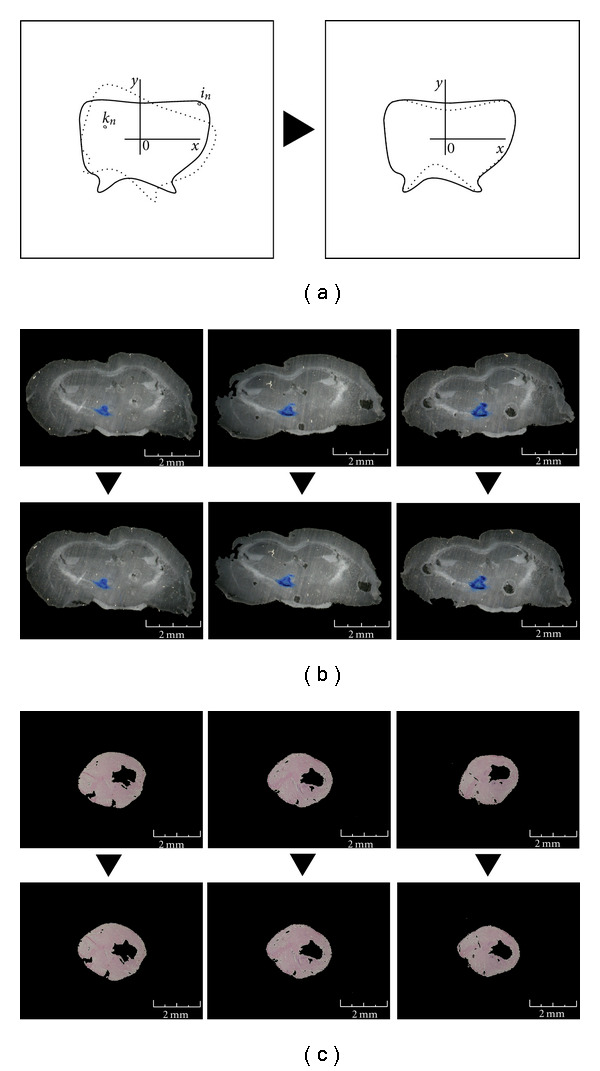
Woven image orientation. (a) Outline of the image orienting routine (see routine 2). The images of the woven area were oriented regarding the adjacent slides by the routine that rotates the woven area of the bitmap 360°, then it chooses the angle where the differences between the adjacent images are the smallest, and hence, the images are the most similar. *i* is the intensity of the pixel number of the first image and *k* is the intensity of the pixel *n* of the previous image. The image chosen is the one with the smallest value of *d*. (b) Example of the image-orienting procedure: In the panels we can see two of the images obtained from the brain serial sections that are rotated with respect to the adjacent image. (c) The same procedure has been used for the heart section images.

The image chosen is the one with the smallest value of “*d*” (Figure 3).

#### 2.6.4. To Reduce Deformation

To reduce image deformation, we used an algorithm that extracts the contour of each section. This algorithm divides the image in an amount of slices depending on the image size. Each slice is a line of pixels from the centre to the edge of image with the concentric lines that can cover the whole 360° degrees of picture. The contour pixels are defined by checking each pixel for each line from the border of picture toward the centre. The first not black pixel is taken as contour pixel. Then, for each angle shot is calculated the distance between the contour pixel and the centre of the bitmap. These distances are annotated for each sequential section. Then, the discontinuity between the sections is diminished by the calculation of the average of the contour pixels with the same angle in the adjacent pictures. The new correct distance of any contour pixel will be

(3) $$L_{n}=\frac{l_{n}+l_{n+1}}{2}.$$

**Figure 4:**
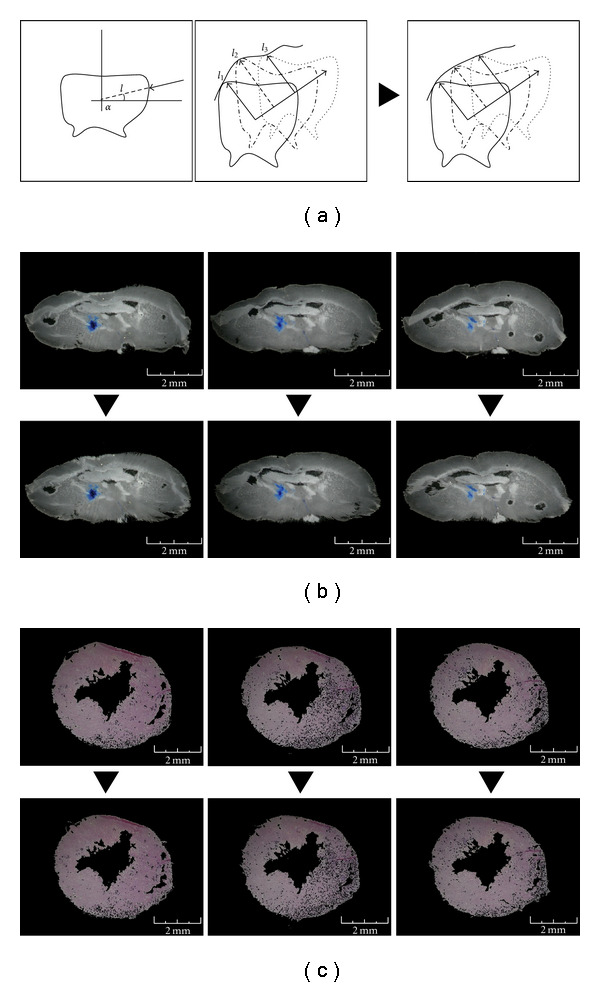
Reduction of the tissue deformation. (a) Outline of the tissue deformation routine (see routine 3). The discontinuity between the sections is diminished by the calculation of the medium of the contours. *l*_*n*_ is the distance between the contour pixel and the centre of the bitmap of the *n* serial section for the angle *α*. With the distance of the contour pixels, the internal area pixels of the section are also adjusted by a proportional linear correction. (b) The images represent three original sequential sections of the brain. In the images below, the deformations have been reduced in order to carry out sequential images which are more homogenous. (c) The images represent three original sequential sections of the heart.

“*n*” is the progressive number of the section and “*l*” is the distance between the contour pixel and the centre of the bitmap for the angle “*α*”. With the distance of the contour pixels, the internal area pixels of the section are also adjusted by a proportional linear correction (Figure 4). 

Absence of image contour could be recognized using image from mouse brain atlas atlas that display tissue sections identical to our.

#### 2.6.5. To Reconstruct 3D Model and Calculate Tissue Volume

The model has been assembled in a file containing a three-dimensional matrix. For the correct dimensioning of the model, we need the relative dimensions “*x*” and “*y*” of a pixel and its depth “*z*”. To this aim, we have calculated the depth of the pixels covering the distances between the sections by carrying out the right proportion between the real dimensions of the tissue section and the pixel dimensions of the tissue area in the picture as follows:

(4) $$X_{\mathrm{rel}\;}=Y_{\mathrm{rel}\;}=\frac{L_{w}}{n_{x}}=\frac{H_{w}}{n_{y}},$$

“*X*_*rel* _” and “*Y*_*rel* _” are the relative dimensions (height, width) represented by one pixel, “*L*_*w*_” and “*H*_*w*_” are the effective dimensions of a part of the tissue, and “*n*_*x*_” and “*n*_*y*_” are the number of pixels that represent these parts of tissue.

(5) $$Z_{\mathrm{rel}\;}=X_{\mathrm{rel}\;}\cdot d_{\text{s}},$$

“*Z*_*rel* _” is the real depth represented by one pixel and “*d*_s_” is the effective distance between two sequential sections. 

The measure of volume has been carried out by calculating each not black voxel (i.e., every voxel representing a part of tissue). 

To visualize and navigate the 3D models, we loaded the files containing data in software allowing the volume visualization of 3D medical images. The software used is the demo version of VolView 2.0 produced by Kitware.

We did not perform data interpolation. In our experimental condition, the isointerpolation 3D volume doesn't increase the precision since the error order of the interpolation of the volume is much lower than the order error occurring during the reconstruction of the model from dissected slice sections.

#### 2.6.6. Isolation of a Molecular Signal into a 3D Model

In order to view only the pixels that represent the *β*-Galactosidase (X-Gal = blue signal), we selected the areas where the blue component of the intensity RGB of the pixels are greater than the other components for a value defined by the customer.

## 3. Results and Discussion

We developed a computational procedure for the generation of a 3D model starting from *postmortem* tissues/organs serial slices in which it is possible to analyze and highlight target molecular signals as well as anatomical areas of interest. 

### 3.1. Routines Development

The total numbers of serial slices processed were 113 for the brain and 100 for each heart, taken from coronal brain serial sections of treated mice and heart serial sections of wild-type mice. Before building the 3D model, we have optimized procedures consisting of four steps.

(i) The first manipulation consisted of soil and glare deletion. The intensity of the pixels outside of the woven area in the slides was selected, and these pixels were transformed into black colour. All sections with strong background noise were excluded from the analysis. However, salt/pepper noise may be eliminated through the selection of colour levels adequately (Figure 1). 

(ii) In the second step, images of each serial slices were centred with respect to the total area of the bitmap. The routine calculates the barycentre of the section area in the image and moves it to the centre of the bitmap (Figure 2). This step generates a compacted model that allows the alignment of the molecular signal (e.g., X-Gal marks within the brain model) and the accurate reconstruction of the tissues/organs (e.g., measure of the heart volume). 

(iii) In the third step, each image from tissue slices was oriented in comparison with the adjacent image by a routine that rotates the section area of the bitmap 360°. For each fraction of rotation, the application calculates and memorizes the summary of the difference between the intensity of each pixel in the image with each pixel in the adjacent image. Finally, the routine chooses the image in which the tissue area is oriented with the best angle for the alignment between the tissues in the adjacent images, that is, the angle with the smallest summary memorized (Figure 3). These elaborations were necessary in order to eliminate the inaccuracy generated during the phase of fixing brain or heart slice sections.

To evaluate the alignment/rotation error, we performed comparative analyses, elaborating brain sections acquired by MRI. The images were downloaded by “The centre of translational neuroimaging” web site, and the model downloaded was “3D MRI Digital Atlas database of an adult C57BL/6J mouse brain” (http://www.bnl.gov/CTN/mouse/). The tissues in the images scanned by MRI were moved and rotated randomly, then, using our application, we have processed these pictures in order to match them with originals. 

Comparative analysis indicated that the inaccuracy was just limited to the vertical curvature of the posterior portion of brain that unaffected the correct reconstruction of the model, even if small error occurs, those errors could at least generate tiny modification of the volume shape but did not interfere with the analysis. 

Thus, our application allowed maintaining the structure of the brain with the attack of the spine. 

(iv) The last step was the correction of the deformation caused by the technical slices preparation. Based on the anatomical structure of tissues, the sections deformations were reduced by the comparison of each section with the adjacent sections. Notably, our method allows a correct analysis of data even tissue/organ are not symmetric. As reported above, modification of the volume (such as shape distortion) did not interfere with the analysis.

 In respect to the active contour model [23, 24], the algorithm that we used extracted the contour of each section and memorized for each degree the distance between the centre and the first not null-pixel by the control from the outside towards the centre of the bitmap. This procedure was replicated for each image. In this way, the surface of model, composed by the contours of the serial images, is memorized and can be smoothed by calculating the average of the contours (Figure 4). Moreover, for the correct reconstruction of the models, the distances of the sections compared to the width of the pixel were calculated.

### 3.2. Assembly and Model Navigation

**Figure 5:**
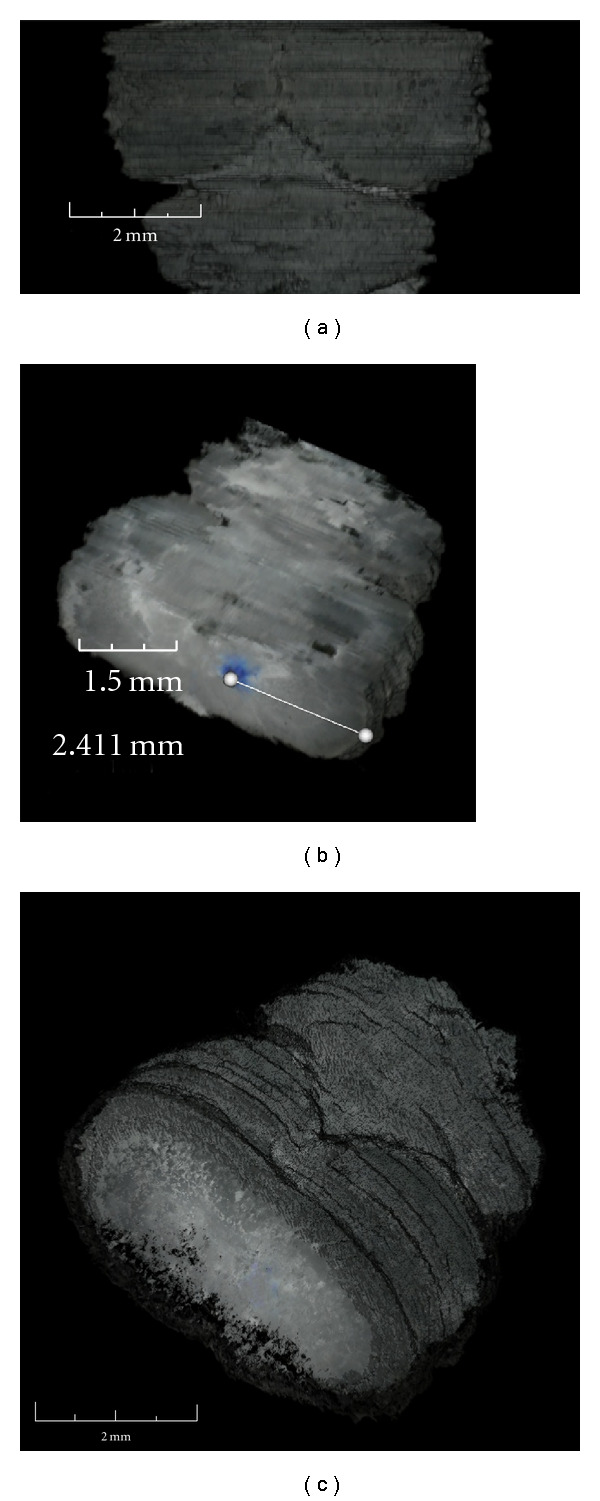
3D views of the brain model. (a) Superior view of the model. (b) Isometric view of the horizontal section of the brain model. In this image we can see the internal structure of the mouse brain and the distance from the external of the brain to the injection point. (c) Isometric view of the full model.

The 3D model can be visualized and highly manipulated by a volume visualization software. It can be magnified, moved, and turned on three axes. The zones of greater interest can be isolated, measured, and analyzed at molecular level (Figure 5). 

Here, we report two different examples of this computational procedure. The first consists in the generation of a 3D brain model using C57/BL6 mice injected with HSV-T0Z herpes simplex viral vector. The second consists in the generation a 3D model of mouse heart. We have used this model to measure the heart volume value. 

### 3.3. Generation of 3D Model of Mouse Brain after Gene Transfer Approach

We used HSV-T0Z, a nonreplicating herpes simplex viral vector reporting the *β*-Galactosidase gene [22, 25]. HSV-1 has the ability to infect a wide variety of cell types in the nonreplicating phase, for example, neurons, as well as the intrinsic capacity to be transported in a retrograde manner to motor and/or sensory neuronal cell bodies following peripheral inoculation [22, 25, 26]. We injected the vector into the internal capsule of the left brain hemisphere. We designed an experimental plan composed of two groups of five-month-old mice. Group 1, C57/BL6 + HSV-T0Z, 5 × 10^6^ total PFU; group 2, untreated C57/BL6 mice. The animals were sacrificed after 72 h and 1 month, and the brains were sectioned on serial slices by a cryostat. The slices were analysed by a NIKON-Eclisse-TE2000 microscope equipped with an Olympus F-View camera. The coronal serial slices images were used to compose the 3D virtual brain model as above described (see video S1 and video S2 in Supplementary Material available online at doi:10.1155/2011/236854).

**Figure 6:**
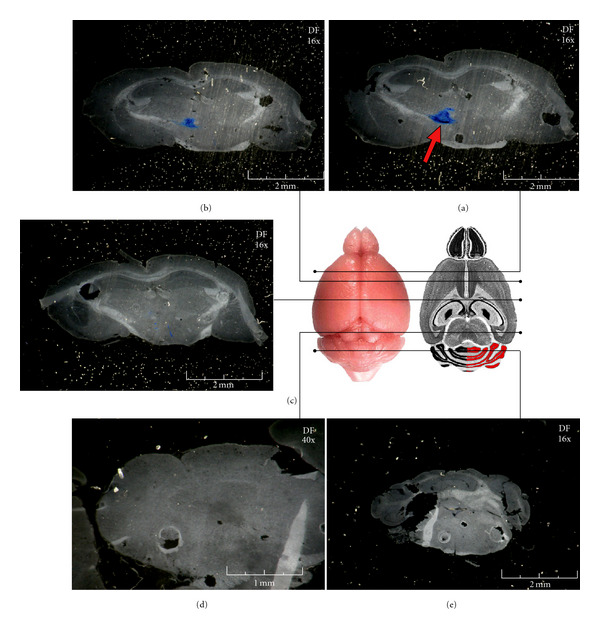
Representative sections of HSV-T0Z distribution in the mouse central nervous system. Serial brain sections were produced dissecting animals in coronal orientations (a–e). Here, we show a part of the representative coronal sections. Sections were stained with the X-Gal substrate (blue signal) as described in method paragraph. In the dark field (DF) images are indicated the magnification and measurement bars. Point of injection is shown (red arrow) into image (a) on the left hemisphere close to bregma line. In (e) there is a representative section of cerebellum.

We analysed the viral vector distribution by monitoring the X-gal staining in coronal, transversal, and sagittal high-throughput brain serial sections. New results confirmed our previous work with a wide viral vector spreading in both injected and uninjected hemispheres (Figure 6). Our computational method allowed a more accurate step-forward analysis of the distribution of those stained proteins. 

**Figure 7:**
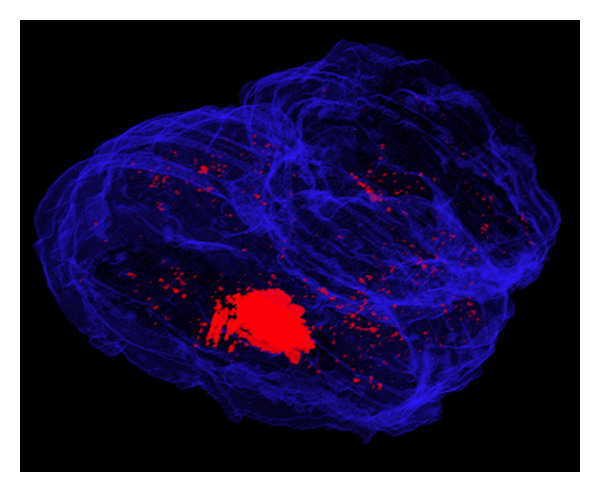
Isolation of the X-Gal staining. An isometric view with the distribution of the X-Gal staining (in red) is shown, highlighting a magnification of the signal. The model has been rendered with a demo version of VolView 2.0 produced by Kitware, USA.

First, we isolated the molecular signal by the selection and the amplification of its levels of tonality (Figure 7). The model allowed the detection of the *β*-Galactosidase stained (the tonality of X-Gal staining is blue and always greater than the general tonality of the brain section which is grey) and the identification of the signal position in all the brain areas. The 3D model allows to appreciate the high distribution of the HSV-T0Z viral vector in brain area far away from the site of injection, in both treated an untreated hemisphere and cerebellum. Notably, the model allowed following the viral vector within the brain after administration over time (Figure 7; video S1). Further, in this model, it is possible to merge the signal with the brain anatomical structure allowing the investigation of putative mechanisms involving the transgenic distribution (video S1, video S2).

### 3.4. Generation of 3D Model of Mouse Heart and Measurement of Tissue Volume

**Figure 8:**
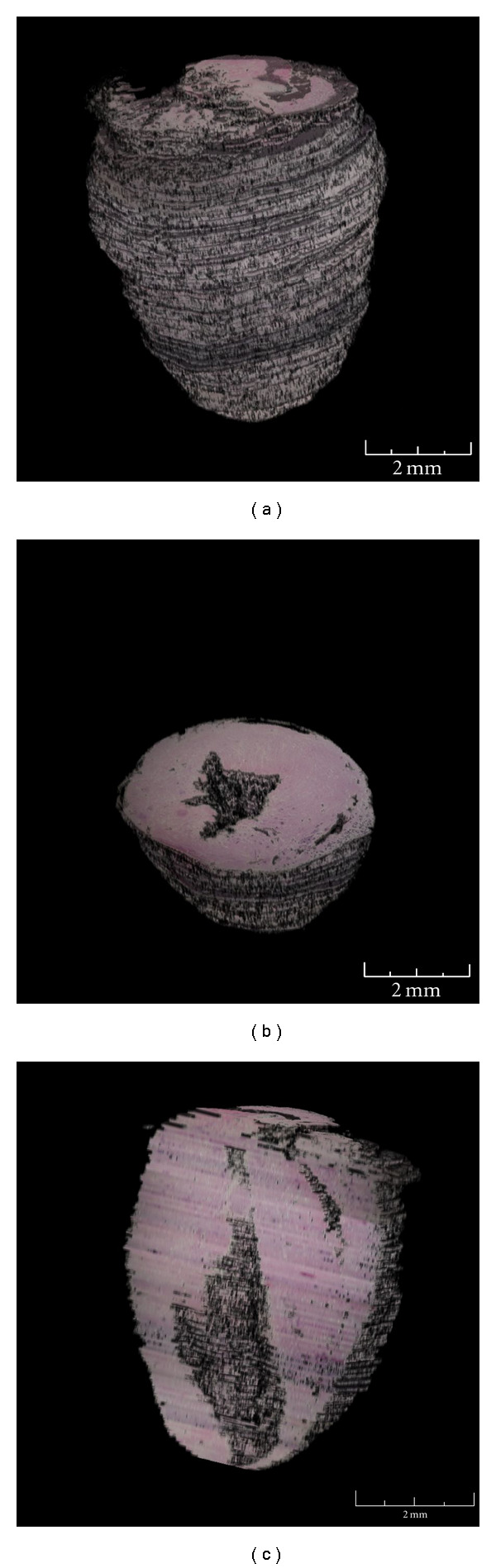
3D views of the heart model. (a) Isometric view of the whole heart model. (b) Isometric view of the horizontal section of the heart model. (c) Isometric view of the vertical section of the heart model. In this image, we can see the ventricles of the mouse heart and the thickness of the heart walls.

To be useful, a 3D model of mouse heart has to recreate the cardiac tissue in physiologic and pathologic state or after therapeutic treatment (i.e., cell transplantation). The current echocardiography is not always comprehensive. Here, we showed that report an example of our method allowed the generation of a 3D mouse heart model (Figure 8) and permitted the measure of its volume. This parameter is particularly relevant for evaluating morphological changes during aging and also for monitoring the clinical progression of cardiac disease in murine animal models [27–29].

The measure of heart tissue volume has been carried out by the calculation of every voxel representing tissue after an accurate evaluation of the virtual voxel volume with respect of real tissue dimensions. We found that the total heart volumes were 65.5 (±0.3) mm^3^ and 84 (±0.4) mm^3^, the total ventricle chamber volumes were 5.2 ± 0.1 mm^3^ and 7.3 ± 0.2 mm^3^, and the total ventricle volumes were 58.3 ± 0.2 mm^3^ and 79.8 ± 0.4 mm^3^, in female and male C57/BL6 mice, respectively. The data were consistent in terms of values, and the difference between male and female values was statistically significant. 

### 3.5. Conclusion

We have developed a new computational procedure able to overcome the limitations of visual analysis emerging by the examination of a molecular signal within images of serial tissues/organs sections by using the conventional techniques (Computerized Tomography, Positron Emission Tomography, Magnetic Resonance Imaging, 3D ultrasound. X-ray, and 3D IR imaging). Despite the high-quality performance in evaluating samples for analytical medicine, these techniques fall on the analysis at molecular level of therapeutics compound (e.g., generic drugs and recombinant proteins). In our case, the method that we developed represents the simplest way to follow the three-dimensional distribution of gene product in an *in vivo* gene transfer approach or to measure volume of an organ such as the heart. The only limitation of this method could be in the state of the original tissue slices. In fact, much damaged brain slices must be discarded. However, due to the availability of adjacent sections to the disrupted slices, the relative loss of data in the final model is minimized.

The 3D position of these molecules as well as their distribution from the site of injection within the tissue/organ architecture represents essential parameters for developing efficacious clinical approaches and, also, for therapeutic drugs design. The main characteristic of our method is in the possibility of having a 3D-imaging apparatus where other classical instruments are not applicable. This procedure is suitable for every condition of imaging analysis postmortem and without particular, sophisticated, or expensive instruments. 

However, on the basis of the easy application of the procedure and mostly for the high-quality information that we obtained, we consider our method to be a simple and valid 3D-modeling instrument for studying the expression and localization of stained cells/proteins/genes/RNA/drugs as well as the tissue architecture, even in the presence of biomaterials for regenerative medicine.

## Supplementary Material

Video 1 and Video 2 showed the 3-D model of C57/BL6 mouse brain after the injection with HSVT0Z herpes simplex viral vector.
The model allowed the detection the *τ*-Galactosidase stained (the tonality of X-Gal staining is blue and always greater than the general tonality of the brain section which is grey) and the identification of the signal position in all the brain areas.
The 3-D model allows to appreciate the high distribution of the HSV-T0Z viral vector in brain area far-away from the site of injection, in both treated and untreated hemisphere and cerebellum (video.1).
Further, in this model it is possible to merge the signal with the brain anatomical structureallowing the investigation of putative mechanisms involving the transgenic distribution (video.1, video.2).
